# How Sensors Might Help Define the External Exposome

**DOI:** 10.3390/ijerph14040434

**Published:** 2017-04-18

**Authors:** Miranda Loh, Dimosthenis Sarigiannis, Alberto Gotti, Spyros Karakitsios, Anjoeka Pronk, Eelco Kuijpers, Isabella Annesi-Maesano, Nour Baiz, Joana Madureira, Eduardo Oliveira Fernandes, Michael Jerrett, John W. Cherrie

**Affiliations:** 1 Institute of Occupational Medicine, Research Avenue North, Edinburgh EH14 4AP, UK; john.cherrie@iom-world.org; 2 Environmental Engineering Laboratory, Department of Chemical Engineering, Aristotle University of Thessaloniki, 54124 Thessaloniki, Greece; denis@eng.auth.gr (D.S.); alberto@eng.auth.gr (A.G.); spyros@eng.auth.gr (S.K.); 3 TNO, Nederlandse Organisatie voor Toegepast Natuurwetenschappelijk, Postbus 360, 3700 AJ Zeist, The Netherlands; anjoeka.pronk@tno.nl (A.P.); eelco.kuijpers@tno.nl (E.K.); 4 Epidemiology of Allergic and Respiratory Diseases Department (EPAR), Sorbonne Universités, UPMC Univ Paris 06, INSERM, Institut Pierre Louis d’Epidémiologie et de Santé Publique (IPLESP UMRS 1136), Medical School Saint-Antoine, F75012 Paris, France; isabella.annesi-maesano@upmc.fr (I.A.-M.); nour.baiz@upmc.fr (N.B.); 5 INEGI, Institute of Science and Innovation in Mechanical Engineering and Industrial Management, University of Porto, Rua Dr. Roberto Frias, 4200-465 Porto, Portugal; jvm@fe.up.pt (J.M.); eof@fe.up.pt (E.O.F.); 6 UCLA Fielding School of Public Health, 650 Charles E. Young Drive South, 56-070B CHS, Los Angeles, CA 90095, USA; mjerrett@ucla.edu; 7 Institute of Biological Chemistry, Biophysics and Bioengineering, Heriot Watt University, Riccarton, Edinburgh EH14 4AS, UK; j.cherrie@hw.ac.uk

**Keywords:** exposome, exposure assessment, exposure factors, sensors, mobile technology

## Abstract

The advent of the exposome concept, the advancement of mobile technology, sensors, and the “internet of things” bring exciting opportunities to exposure science. Smartphone apps, wireless devices, the downsizing of monitoring technologies, along with lower costs for such equipment makes it possible for various aspects of exposure to be measured more easily and frequently. We discuss possibilities and lay out several criteria for using smart technologies for external exposome studies. Smart technologies are evolving quickly, and while they provide great promise for advancing exposure science, many are still in developmental stages and their use in epidemiology and risk studies must be carefully considered. The most useable technologies for exposure studies at this time relate to gathering exposure-factor data, such as location and activities. Development of some environmental sensors (e.g., for some air pollutants, noise, UV) is moving towards making the use of these more reliable and accessible to research studies. The possibility of accessing such an unprecedented amount of personal data also comes with various limitations and challenges, which are discussed. The advantage of improving the collection of long term exposure factor data is that this can be combined with more “traditional” measurement data to model exposures to numerous environmental factors.

## 1. Introduction

Most non-communicable diseases are a result of a complex combination of genetic susceptibilities and environmental exposure, however, environmental factors have not been comprehensively considered in disease aetiology at the individual level [1]. The exposome represents the totality of human exposures from conception to death [2,3]. It is, therefore, both longitudinal and multi-faceted. A major challenge in the coming decades will be to better define how the combination of the genome and exposome contribute to the risk of disease.

**Figure 1:**
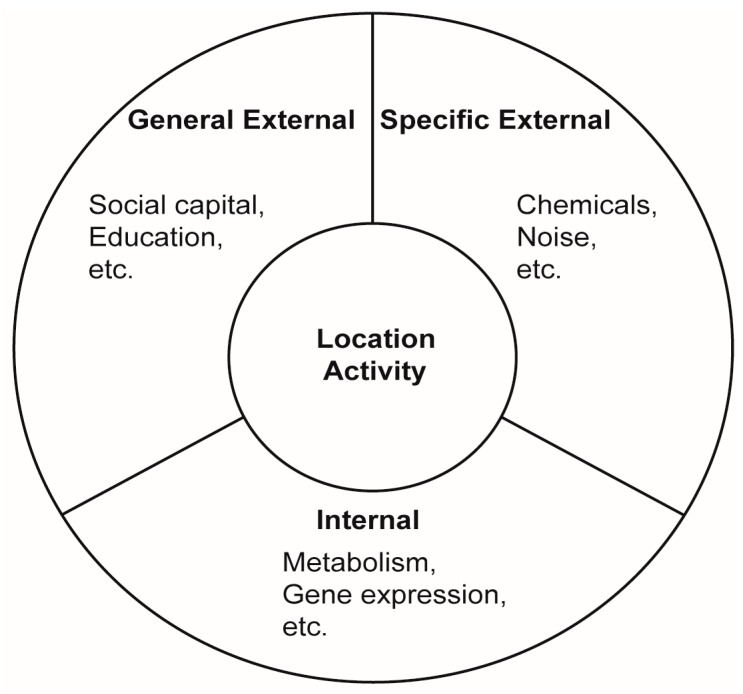
Adaptation of the three spheres of the exposome, as defined by Wild [4], showing that assessing a person’s location and activity patterns influences all areas of the exposome.

Wild [4] defines three broad and related spheres in the exposome: internal, general external, and specific external (Figure 1). Ideally, quantification of the external exposome would include all pathways of external exposure (e.g., inhalation, ingestion, and dermal contact) and relevant stressors, such as air pollutants, contaminants in food and water, soil and dust, radiation, temperature, light, and noise. In addition, exposure factors (e.g., inhalation rate, consumption amounts and frequencies, and location) should be characterised. Physical, social, and individual factors such as diet, physical activity, and stress are also important when accounting for the relationship between environment and health. This breadth of exposures and risk factors vary over many temporal and spatial scales, creating an extensive and challenging task for monitoring.

As personal smart technologies become more prevalent there is the promise of portable, lower cost sensor systems that would enable wider-scale and longer-term monitoring of either personal exposure or the external environment close to the person. The increasing popularity of small sensors, open-source programs, microcontrollers, wireless technology, and cloud computing has allowed for the collection and integration of many types of personal level data. There is even a “quantified self” movement, a community of people interested in using technology to gather data such as location, activities, diet, sleep, etc. in the interest of better understanding one’s own habits and their impact on their health. Such a “quantified self” would be of interest for exposome research. The question then, is what technologies may best serve researcher needs and how can these best be incorporated into studies?

A central concept that we propose for using sensors in external exposome assessment is in assessing location and activities longitudinally (Figure 1), which are key influencing factors for all three spheres in the exposome [5,6]. Location determines the amount of a hazard present and behavior determines duration of exposure to a hazard (e.g., air pollution). Location is an important determinant of the social environment of the individual, the climate, and other aspects of the general external exposome. Furthermore, location and behavior (including diet) can influence a person’s microbiome, and other aspects of their internal exposome [7]. The concept of assessing location and matching this with environmental data collected from either other instruments or sources is fundamental to exposure science. Furthermore, sensors may help push the frontier with their potential to allow for collection of detailed location and activity data over a much longer term than typically done in exposure studies. In effect, this is a “time-geographical” approach [8,9,10], using the tracking of daily location (via phone Global Positioning System (GPS)), long-term location (via questionnaire on residential and job history), and activities and behaviour (via questionnaires, sensors, and smartphone apps). These can serve as a basis for developing models which will help us examine the factors in a person’s physical and social environment that influence their exposome. In this manuscript, we explore features of sensors which would facilitate their use in this “time-geographical” approach.

## 2. Sensor Criteria

Most personal exposure studies require participants to carry a backpack or bag that holds monitoring equipment as they go about their daily activities [11,12,13,14]. This limits the duration for which exposures can be assessed, and requires participants to carry an often heavy and possibly noisy package around with them. Due to expense and logistics, detailed exposure studies have often been limited in the time scale covered. For example, personal air pollution studies were often panels of a few tens of participants, and limited to 24–48 h of monitoring at a time. Similarly, dietary exposure studies also captured a relatively small window of time. If we want to cover the breadth of the exposome—not only in terms of number of exposures but also length—we must find a way to incorporate monitoring into everyday life.

There is a wide range of research development of sensors, apps, and other smart technologies for mobile health and environmental monitoring. Research instruments, however, often suffer from a lack of friendly user interfaces. As we are interested in longer term monitoring, we examine the feasibility of using “off-the-shelf” commercial devices aimed at the lay person in a European context, to integrate monitoring more effectively into a person’s lifestyle. These instruments are often less costly than research grade instruments, and therefore allow for the possibility for greater numbers to be deployed. We define several criteria that should be met by sensor and smart technologies:

1. Unobtrusive to the user. Unobtrusive means that the item is easily worn/carried/placed.
2. “Cost-effective”, i.e., such that widespread deployment of sensors is a practical proposition for the purpose and context of the study. Most research grade instruments are too expensive for large scale personal monitoring, either due to the expense of the equipment, and/or the cost of proprietary software licenses.
3. Able to collect, store and transmit real-time and high temporal resolution data.
4. Useable by a lay person.
5. Ability to connect to the internet so that collected data can be remotely accessed by researchers and users, or, the ability to store collected data for download.
6. Meets predefined quality assurance and quality control (QA/QC) specifications as defined for devices of a particular type, including: (i) Sufficient sensitivity and specificity or detection limits that allow environmental concentrations or other factors to be measured; (ii) Low failure rate; (iii) Adequate precision and accuracy to assess the relevant exposure; and (iv) Stability over time.

The last criterion requires further definition as to our tolerance. Every instrument entails some degree of error in terms of accuracy and precision. Reference instruments for regulatory monitoring, for example, for air or water quality, have predefined criteria set by governments, and are often considered a gold standard. Researchers must define the tolerance limits for their studies. Any instrument to be deployed in numbers must have demonstrable precision with respect to each other and stability over time. Accuracy must be known—any biases should be understood and correctable. However, we must also ask what the purpose of the sensor use is—do we want to just be able to rank people and separate them into differential exposure groups? In that case, sensors need to be able to provide stable relative measurements, but the accuracy of these is less important. On the other hand, do we need to know the exact exposure value? In that case, sensors must be demonstrated to reliably reflect gold standards and to be carefully calibrated. Either way, it is important to clearly characterise the errors and uncertainties entailed in using sensor instruments.

## 3. Exposure Parameters and Sensors

Several exposure-related parameters lend themselves especially well to evaluation using consumer smart technologies. They are generally parameters that people have a personal interest in logging, and there has been enough market appeal for there to be several devices available to the public. These include location, physical activity, diet, and indoor environmental quality. There is also a large and growing interest in air quality sensors, both by researchers and the general public, particularly in urban areas. We discuss these briefly.

### 3.1. Location

Personal location data can be collected using a GPS device or a GPS‑enabled mobile phone. The information provided by a GPS can reduce personal exposure measurement error [15,16], which could lead to a substantial misclassification of exposure to environmental factors.

In terms of our six sensor selection criteria, GPS units and mobile phones are generally high scoring in terms of being portable and unobtrusive. Although GPS units suffer from data loss or lack of accuracy when signals are lost or reflected due to their presence in concrete and steel buildings or among many high rise buildings, signal losses have been used as a means of determining whether a person is in- or outdoors [17,18]. The accuracy of GPS units tends to be in the range of a 5 m or greater buffer, and data need to be processed before they can be used in exposure studies [18,19,20].

GPS-enabled mobile phones have also been found to be acceptable to subjects as a means of location tracking and display similar tracking capabilities to non-phone GPS units [18,21,22]. Smartphone location apps are generally free for those who already own such a phone. Mobile phones have an added advantage that most people carry them anyway, and therefore there is no need for an additional instrument. Smartphone apps allow for remote access, thus facilitating the ease of collection by researchers. Location data collected by smartphones, however, may have greater issues with data loss (e.g., from phones being off or not being carried) [20] and accuracy [22]. Tracking apps have also been developed for research studies, and demonstrated as successful means of logging location and activities [23,24]. Smartphone location apps are particularly popular for tracking exercise, however these are not as feasible for exposure studies, due to the drain on battery life from the constant use of the GPS. On the other hand, background tracking apps such as MOVES and Google location services read location at periodic intervals, also using internet wireless signals to gauge location. These have been relatively successful when used in exposure studies [23,25].

In addition to geographic location, presence indoors, outdoors, and in-transit is of particular importance for exposure. Indoor/outdoor presence may also be estimated using data classification algorithms [26,27,28]. GPS units also measure velocity, allowing assessment of transportation mode with reasonably high accuracy (>95%), depending on the velocity classification parameters chosen [27,28]. Phone apps can also estimate transportation mode, although there has not been a rigorous evaluation of accuracy for these activity apportionments of smartphone apps. Various studies have used measurement of additional variables such as temperature and light to attempt to improve GPS location data and indoor/outdoor presence. Although, the use of temperature needs to be examined across a wide range of climate settings and it is not certain that additional concurrently measured variables add much value to the classification accuracy [17,27,28]. In addition, the recently developed Indoor/Outdoor Detector (IO Detector) for Android operating systems uses three types of lightweight smartphone sensors (i.e., light sensor, cellular module, and magnetism sensor) to detect whether one is indoors, outdoors, or semi-outdoors (e.g., in a covered outdoor area). Developers showed a prompt and accurate detection in various time and environments [29]. However, others have found an accuracy of as low as 35%, due to difficulty with new environments or use on devices not used during the training period, which may be improved using a supervised machine learning method [30]. At this time, there has not been a demonstrably reliable means of apportioning indoors/outdoors presence beyond the use of classification models for GPS data collected by either stand-alone units or phones as described above. The collected location data can be matched with pollution data to better determine predictors of personal exposure. For example, this can be used in tandem with a personal monitor, or, through modelling, can reconstruct a person’s exposure [20,31,32].

### 3.2. Physical Activity

Physical activity is both a risk factor for disease and modifier of environmental exposures. Measuring physical activity is important for estimating exposure variables such as inhalation and metabolism. Measures of energy expenditure can be used to estimate breathing rates, which can then be used to estimate inhalation of air pollutants. Additionally, lack of activity may be linked to risk factors (e.g., obesity or diabetes) which can modify environmental impacts on health. There are numerous types of physical activity sensors available, both designed for research purposes and for public consumption [33,34]. These sensors are generally based on triaxial accelerometers, which measure acceleration along three orthogonal axes, and can be complemented by gyroscopes, which measure angular motion. Measurement around several axes allows estimation of both movement and posture. Sensors may be worn on a single area of the body (e.g., wrist or waist), or on several areas of the body. The waist is often a default location as it is close to the centre of mass of the body, although for constant long-term use, the wrist may be more convenient for the user. The accelerometer output needs to be transformed into a meaningful unit for interpretation, such as steps or metabolic equivalents (METS). The activity counts recorded by the accelerometer can be related to energy expenditure using regression equations or other models [33]. Several types of activity sensors have been developed for use in research studies. The Actigraph instruments (ActiGraph, Pensacola, FL, USA), has been used in various studies, including in the US National Human and Nutrition Examination Survey (NHANES) [35]. IDEEA^®^ (MiniSun, LLC, Fresno, CA, USA) and SenseWear (BodyMedia, Pittsburgh, PA, USA) also have been used in population studies [34]. CalFit, a software application for Android systems, was used in Barcelona (Spain) to estimate inhalation doses [20,23]. Sensors are also of interest in sport performance, and have been used to measure heart rate, R–R interval, breathing rate, posture, activity level, acceleration, speed, distance, and location [36].

Commercially available activity monitors are becoming more common for people interested in tracking their activity, sleep, and diet, providing motivation to live a healthier lifestyle. Some of these have been evaluated against “gold standard”, and/or research grade or research grade activity monitors [33,34,35].

Evaluations of a number of fitness trackers, including several models of Fitbit (San Francisco, CA, USA), Jawbone (San Francisco, CA, USA), Nike Fuelband (Nike, Beaverton, OR, USA), Withings Pulse (Withings, Nokia, Issy-les-Moulineaux, France), Basis Peak (Basis Science Inc., San Francisco, CA, USA), Microsoft Band (Microsoft Corp., Redmond, WA, USA), and Apple Watch (Apple, Cupertino, CA, USA) have found varying degrees of concordance with either research grade trackers (e.g., ActiGraph or Actical (Philips, The Netherlands)) or other reference methods of energy expenditure such as indirect calorimetry or doubly labelled water [37,38,39,40,41,42,43,44,45]. Fitbit, for example, an extremely popular fitness tracker, has been found to have low percent error compared with reference methods for energy expenditure estimation and step counting in some studies [39,45,46,47], but could be more biased in others [40].

Additionally, smartphone apps are available that can track a person’s location using the phone’s GPS system along with movement via the phone’s accelerometer. Phone apps alone, however, require the user to carry the phone with them at all times, the app be switched on at all times, often with GPS and Bluetooth active, all of which increase battery drain. User behaviour, such as forgetting to carry their phone with them or not charging it, may result in loss of exposure data [20]. Use of a separate fitness tracker is recommended, and wrist-based ones are generally more convenient for the wearer than body worn ones. If respiration is of particular interest, an activity sensor that is specifically calibrated to respiration may be more useful [36,48,49].

### 3.3. Diet

Computerized questionnaires, especially online and on mobile devices, have an added advantage of convenience for the user, as they may complete the questionnaires after meals or at some other convenient time. Many smartphone apps have been developed as tools for people to track their dietary intake for weight loss or fitness purposes [50]. However, little validation of these apps for estimating dietary intake or related parameters such as energy or nutrient intake has been undertaken [51]. Epidemiology studies have used food frequency questionnaires (FFQ), 24 h dietary recall (24-HDR), and dietary records (DR) to assess people’s food intake. A combination of FFQ and one of the latter methods have been found to be feasible options for epidemiology studies [52,53]. Studies have also used online questionnaires as a means of gathering data, with somewhat mixed but promising results for the use of web-based FFQ or 24-HDR for assessing dietary data compared with non-web-based methods [54,55,56,57,58,59]. Dietary assessments are laborious for participants, but more passive methods, such as wearable cameras, have not yet successfully been implemented on a wide scale. More work is needed to understand the utility of diet apps for exposure assessment needs.

### 3.4. Indoor Climate

Several smart and wireless sensor based devices, e.g., NetAtmo (NetAtmo, Boulogne-Billancourt, France), Foobot (Airboxlab, San Francisco, CA, USA) are available for people to monitor their indoor climate, allowing them to access their home data remotely. Various aspects of the indoor environment influence the comfort of residents and are predictors of levels of indoor stressors such as air quality, dampness, and mould. These parameters include temperature, relative humidity, and ventilation rate. Temperature in itself is a risk factor, and the home environment plays a moderating role when it comes to extreme temperature events. Relative humidity and temperature are important determinants of dust mite, mould and other microbial growth, and also impact the chemical reactions that occur indoors to produce secondary pollutants (e.g., formaldehyde). Ventilation rates can be used to mechanistically model the levels of indoor pollutants derived from indoor source emissions and infiltration from the outdoors. The latter can be approximated using carbon dioxide measurements.

### 3.5. Air Quality

In recent years, there has been a lot of interest in a new generation of air sensors, which show promise in their small size, ability to monitor in real time, and relatively lower cost than reference methods. They have been explored as a means of improving the spatial resolution of air pollution data beyond the existing monitoring network and for personal sampling [60,61]. At this time, however, there are still issues of quality assurance for many of these devices when used in the field, particularly for mobile or personal monitoring [62]. Gas sensors, in particular suffer from cross sensitivities and interferences by external conditions such as humidity, and can be subject to drift and decreased sensitivity over time [63]. The most commonly used ambient gas sensors at this time are metal oxide (MOS) and electrochemical (EC) sensors, available for carbon monoxide (CO), ozone (O_3_), nitrogen dioxide (NO_2_), ammonia, hydrogen sulphide, and total volatile organic compounds (TVOCs). In MOS sensors, gas diffuses into the porous material, changing the conductivity upon reaction with an oxidizing or reducing gas [64]. This change can be measured and related to the gas concentration. Humidity can interfere with the sensitivity of metal oxide sensors [65] and air temperature can also affect the sensor response. In electrochemical cells, reaction with a gas produces an electrical current that is proportional to the gas concentration. These sensors also suffer from cross sensitivities to other gases; in particular, NO_2_, while EC sensors can be quite sensitive to ozone interference. Ozone scrubbers or applying a correction factor for NO_2_ using O_3_ are potential solutions to this interference [61,66]. Sensors’ performances can be quite variable between instruments and their long-term reliability is unknown. Sensitivity to rapid changes in temperature and humidity make MOS and EC sensors more suitable to stationary area monitoring, rather than mobile personal monitoring, where movement between indoors and outdoors can result in fast changes in these conditions. Although temperature and humidity corrections could be done, these must be measured such that the response times correspond with the gas sensors.

Particulate matter sensors, on the other hand, may provide more promise for the current day, considering that these usually use an optical method, including a laser in higher-end sensors. This is similar to the optical methods used by many research-grade sensors. Particle sensors generally count the number of individual particles passing through a sensing volume using light scattering or some other metric, such as the time particles are detected in the sensing volume. The light source is either provided by a photodiode or a laser, with the former being less sensitive [67]. These instruments may be effective for indoor monitoring, particularly when a strong source is present, such as cigarette smoke [68,69]. Conversion of the particle counts into a concentration of mass per volume requires either calibration with a gravimetric device, or estimation using assumed particle density. Some particle sensing devices, such as the Dylos (Riverside, CA, USA), have been demonstrated as having equivalent utility to a conventional device of a similar sensing mechanism [69,70]. The Dylos is commonly used in exposure studies, thanks to its relative cost-effectiveness, reasonable QA/QC and ease of use. However, it is limited in the amount of data it can store and is not remotely accessible without customization. Numerous particle sensors using an optical particle counter are growing in popularity, but costs are still relatively high once development costs are considered. A recent development of a relatively inexpensive integrated particle sampler [71] is another possibility although it does not provide real-time sensing as optical methods do. At this time, however, air sensing devices are more likely to be conducive to stationary than portable monitoring, although advances are being made in the personal monitoring sector, where monitoring over several months has been demonstrated in a study of chronic obstructive pulmonary disease (COPD) patients [72]. Costs are still relatively high for air sensing devices compared to the other types of sensors described in this manuscript (several hundred Euros compared to less than 200 euros or free, in the case of apps).

### 3.6. Noise

Noise levels can be measured in occupational and environmental settings using hand-held sound meters, which measure noise at fixed locations, and noise dosimeters, which are worn on a person to measure personal noise exposure. International standards for both types of instruments are available, specifying performance standards such as frequency weighting requirements and tolerances at various frequencies. Smartphone noise measurement apps are available, many for free, although these may not comply with the standard specifications. Some apps state that most microphones in phones are aligned to the human voice (300–3400 Hz, 40–60 dB) rather than standard specifications, therefore may not effectively measure very loud sounds or sounds outside that frequency spectrum

Due to variability between different brands and models of phones and phone microphones, these apps do not perform similarly with different hardware, especially outside the human voice range [73]. Other issues include a lack of calibration for many versions, although some allow for the user to do their own calibration (e.g., the iPhone apps SPLnFFT Noise Meter and SoundMeter); not all apps use the recommended A-weighting, or provide any weighting options; and they lack frequency analysis capability. Nonetheless, these apps have proved useful for citizen science projects such as Noisetube, which used phones for participatory noise mapping [74]. Smartphone noise apps are not usually calibrated. In an evaluation of 10 iOS apps and four Android apps, Kardous and Shaw [73] found several iPhone apps that met secondary occupational relevancy criteria (according to the American National Standards Institute (ANSI) requirements), while Murphy and King found that iPhone apps tended to measure on average 2.93 dB(A) above generated noise levels in a reverberation room. On the other hand, Android apps were much more variable and tended to under measure by 2.79 dB(A) on average [75]. Kardous and Shaw also found that these apps were consistent between iPhones of the same generation, although they performed differently depending on the microphone type. The variability in performance of Android apps across the different tested phones are likely because Android phones are made by different manufacturers, while iPhones are all made to the same specification. Characterization of noise (e.g., frequency analysis) is missing in most apps. The type of noise (caused by e.g., music, traffic) and people’s noise sensitivity influence potential health outcomes [76]. Another disadvantage is that noise apps on smartphones only allow measurement while the app is in the foreground, and only a few allow exporting of data or calibration with a sound level meter.

## 4. Discussion

There are numerous other potential sensors but we are still far from being able to do a comprehensive monitoring of the external environment with sensing technology. Wearable sensors, mobile apps, and other devices provide new possibilities for assessing aspects of the external exposome, but there are still challenges to be overcome in using these technologies for scientific study. An exposome monitoring system built around a smaller set of sensors, supplemented by data from stationary or publically available sources is much more feasible at this time. Sensors can be placed in homes and other locations, where climate conditions are less variable, to provide information on indoor exposures. Personal exposures can be estimated by integrating various data sets. For example, short-term location data (GPS) on people’s trajectories can be combined with hazard levels at these locations (e.g., air pollution, noise, and UV). Exposure to dietary contaminants or nutrients can be modelled by using the dietary intake data collected via the app with national market basket databases of various food contaminants. Smartphones have been used for Ecological Momentary Assessment (EMA), a method of ascertaining a subject’s current or recent state-of-being, via questions that the subjects are periodically notified to answer [77]. EMA is often used in psychology studies, for example, to study addiction or stress [78].

At this time, sensors are best suited for assessing location and activities, which are key influencing factors for all three spheres in the exposome (Figure 1) [5,6]. They are less well suited to reliably measure actual hazards, such as air pollution or noise, however this may change in the future. Given that sensors are well placed to incorporate monitoring of everyday places visited, movements and habits, and are currently being used by the general public, not just public health study participants, they can provide a useful means of improving long-term estimates of exposure, when used in conjunction with environmental samples or modelled data.

One advantage of data gathering from smartphones and sensor-based technologies is that information from different wireless devices and apps can be gathered in the field and stored with less need for researcher intervention, unlike conventional forms of exposure assessment. Most wireless devices which consumers use have an app or webpage from which data can be accessed and viewed or downloaded. Sometimes, the developers of the consumer product provide the user access to the data collected via an application programming interface (API), a set of routines, protocols, and tools for building software applications. The API specifies how software components should interact. If an API is available for an app, this data (if permitted by the data creator, i.e., study participant) can be accessed in an internet portal to securely access and store data. This portal could be a method for data storage and management, but also a possibility for interacting with the participants. The portal also allows researchers to check that data is being uploaded during the study, so if there are any difficulties it is possible to contact the participants during the study period to remind them to collect/upload data, or check how things are operating. This helps reduce the potential for loss of data.

Another key issue in sensor based exposome studies is related to storage, processing, and analysis of the huge amount of data which can be collected with these sensors with long term deployment. Considering the multitude of exposures to be measured to characterise the exposome, it is critical to have a system that can store, manage, and integrate a large amount of data. The data acquired by individual sensors will need processing and interpretation (e.g., human behavior recognition). This requires statistical advances, sophisticated data mining techniques, computing power, and a careful sharing of data sources while also maintaining privacy protection for personal data. Without significant advances in this area, the analysis of the wealth of data threatens to become the new limiting factor in further progress [79].

At this time, sensors are unlikely to meet reference instrument or “gold standard” criteria, but it is possible to set secondary data quality objectives that are more easily attainable, which allows sensors to be used as complementary data collectors and thereby increase data quantity. A key question that needs to be answered in this context is what is the overall uncertainty introduced by the use of ubiquitous personal sensors, against the uncertainty resulting from the temporally and spatially deficient regulatory monitoring networks?

The use of wireless devices and storage of information on the internet also leads to potential security concerns. Furthermore, privacy and ethical issues are raised when these technologies are applied for assessing exposure to environmental stressors. Issues of data ownership and data protection need to be clarified and structured to allow ubiquitous environmental health monitoring to become an everyday reality. Many devices and apps identified in this article are available to the public, and people must agree to terms and conditions regarding their privacy associated with use of these products. By participating in a study using such items, they are also agreeing to the same terms and conditions, along with agreement to provide researchers access to the information collected. Researchers must make these conditions clear to the participants as part of the informed consent process, and note that a participant not only agrees to allow researchers access to their information, but that the company whose product is being used may also have access to the same data. In the “world of data ownership” much is still unclear. Companies are generally able to decide how they wish to deal with data privacy, without consulting users. Data protection laws vary between countries, but the data collected by apps and smart devices are international. European studies, for example, may want to ensure that devices used comply with European data protection laws or use devices whose data are stored in Europe rather than the United States. There is also a trend of larger companies purchasing smaller ones, which means that data ownership may change, although the consumer may not always be aware of such changes. Furthermore, there is the possibility that sensor-gathered data could be requested for use in court proceedings or by insurance companies [80]. These issues should be considered in the selection of devices and apps for studies or the development of apps specifically for studies. In particular, when using a third party device, researchers must be aware and understand the details of device privacy policies so that they can inform their participants. Wherever possible, the least amount of personal data needed should be provided, anonymous user accounts used, and the account should be deleted after a defined amount of time. The latter request may need to be directly made to the company which makes the device, although researchers need to make participants aware that sometimes companies may delete an account but retain the right to use “anonymised” data [81].

Researchers are required to ensure safe storage and encryption of participant data. However, one of the advantages we mentioned with sensors and apps is the use of cloud computing. Data are relayed via Bluetooth to an internet enabled device (e.g., smartphone or tablet) and stored via the cloud. Most sensors developed for the public market do not have on-board storage and are reliant on cloud storage. Secure peer-to-peer encryption is needed for signal transmission, and the databases must be securely encrypted. Passwords given to participant accounts should also have a certain level of complexity to ensure security. Still, unless researchers develop and manage the devices and data collected, they can only control the security of downloaded data, and those collected by devices such as Fitbit or NetAtmo will be subject to the data owners’ security procedures. As with all wireless devices and cloud storage, there are concerns that unauthorized parties may hack into systems and access the data. Again, researchers will need to consider all these issues when deciding to use smart technologies, using appropriate safeguards, and informing participants. Harmonization of data handling and standardization of data privacy and confidentiality procedures are needed to support the wider use of personal sensors in this context.

## 5. Conclusions

The exposome, or the “totality” of a person’s lifetime exposure, is a vast concept which exposure science is still trying to resolve. The rapid advance of sensing and wireless technology has opened up a new and exciting frontier for exposure science, by providing means to measure across time and space, and perhaps in the future across many different aspects of the exposome. These possibilities also open up new questions as to whether and how sensor based data can be used for better understanding the health impacts of the environment, while assuring the privacy of the participants. There are still challenges for sensing devices in terms of data quality, form and function, cost, management, and analysis of the amount of data that could potentially be collected. As research and development progress, scientists, product developers, computer scientists, human interaction designers, and ethicists will need to work together to push forward and manage this era of sensor technology and big data. In the meantime, exposome projects will need to balance the possibilities of new sensor technologies with more conventional and well-tested methods of data collection. A practical approach is to document spatial time–activity profiles of subjects, and to integrate these data with other existing datasets, such as air and water quality, to generate modelled estimates of a person’s external exposome.
